# Simvastatin Efficiently Reduces Levels of Alzheimer’s Amyloid Beta in Yeast

**DOI:** 10.3390/ijms20143531

**Published:** 2019-07-19

**Authors:** Sudip Dhakal, Mishal Subhan, Joshua M. Fraser, Kenneth Gardiner, Ian Macreadie

**Affiliations:** School of Science, RMIT University, Bundoora, Victoria 3083, Australia

**Keywords:** Alzheimer’s disease, amyloid beta, ageing, autophagy, proteostasis, statins, yeast

## Abstract

A large-scale epidemiology study on statins previously showed that simvastatin was unique among statins in reducing the incidence of dementia. Since amyloid beta (Aβ42) is the protein that is most associated with Alzheimer’s disease, this study has focused on how simvastatin influences the turnover of native Aβ42 and Aβ42 fused with green fluorescent protein (GFP), in the simplest eukaryotic model organism, *Saccharomyces cerevisiae*. Previous studies have established that yeast constitutively producing Aβ42 fused to GFP offer a convenient means of analyzing yeast cellular responses to Aβ42. Young cells clear the GFP fusion protein and do not have green fluorescence while the older population of cells retains the fusion protein and exhibits green fluorescence, offering a fast and convenient means of studying factors that affect Aβ42 turnover. In this study the proportion of cells having GFP fused to Aβ after exposure to simvastatin, atorvastatin and lovastatin was analyzed by flow cytometry. Simvastatin effectively reduced levels of the cellular Aβ42 protein in a dose-dependent manner. Simvastatin promoted the greatest reduction as compared to the other two statins. A comparison with fluconazole, which targets that same pathway of ergosterol synthesis, suggests that effects on ergosterol synthesis do not account for the reduced amounts of Aβ42 fused to GFP. The levels of native Aβ42 following treated with simvastatin were also examined using a more laborious approach, quantitative MALDI TOF mass spectrometry. Simvastatin efficiently reduced levels of native Aβ42 from the population. This work indicates a novel action of simvastatin in reducing levels of Aβ42 providing new insights into how simvastatin exerts its neuroprotective role. We hypothesize that this reduction may be due to protein clearance.

## 1. Introduction

**Figure 1:**
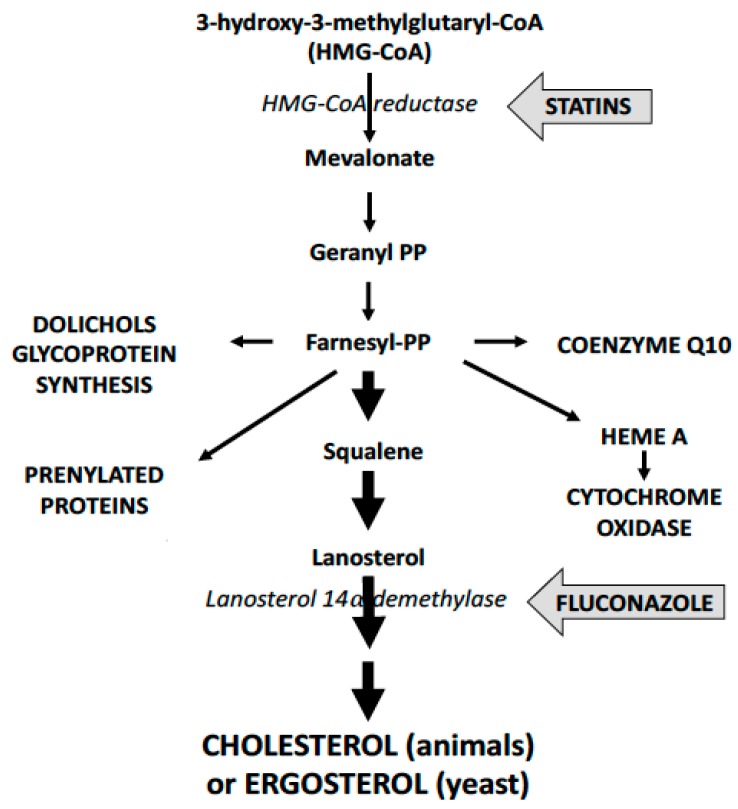
Simplified schematic view of cholesterol and ergosterol biosynthesis pathway in animals and yeast. Statins target the highly conserved catalytic site of HMGCR to inhibiting of cholesterol and ergosterol synthesis. Azoles, like fluconazole, target fungal lanosterol 14α demethylase to inhibit ergosterol synthesis.

Statins are highly effective, widely-prescribed drugs that bind to and inhibit HMG-CoA reductase (HMGCR) reducing the liver’s synthesis of cholesterol (see Figure 1, cholesterol biosynthesis pathway). An unpredicted effect was found in a large-scale epidemiology study: simvastatin halved the incidence of Alzheimer’s Disease while there was no similar effect in users of atorvastatin or lovastatin [1,2]. 

There has been interest in understanding how simvastatin might exert this effect. Indeed, simvastatin is the most lipophilic statin, and this might enable greater uptake in the brain where 20% of the body’s cholesterol is synthesised, however, to our knowledge there have been no studies on the relative uptake of statins in human brains. The next question relates to the mechanism of simvastatin action, and some studies have looked at the effects of statins on Aβ42 production. As can be seen in Figure 1, inhibition of HMGCR affects some additional pathways, including protein prenylation [3]. One of the proteases leading to the production of Aβ42, BACE, is a prenylated protein. Studies of mammalian cells in culture show that numerous statins inhibit BACE prenylation resulting in less Aβ42 production [4]. In the brain simvastatin may be inhibiting Aβ42 production. 

The important and integral part of cell membranes that helps in cell regulation, maintenance of fluidity and provides stability to cell is cholesterol [5]. Microdomains (lipid rafts) present in the membrane have small patches of various lipids including cholesterol which is thought to be involved in pathophysiology of AD [6]. Most of the enzymes responsible for the transmembrane APP cleavage, such as β- and γ-secretase, function optimally in a cholesterol-rich environment. The activities of these enzymes are related to the concentration of cholesterol in lipid rafts [7,8,9]. The link between the cholesterol and Aβ42 deposition has been elucidated by studies using transgenic mice. Brains of APP-transgenic mice showed increased levels of Aβ42 deposition when fed on a diet rich in cholesterol [10,11].

In human studies, the treatment of hypocholesteraemia in post-menopausal women by statins decreased the prevalence of AD, showing that cholesterol synthesis and AD are interlinked [12]. Treatment of AD patients with lovastatin showed a decrease in Aβ42 serum levels [13] while atorvastatin has been reported to decrease the cognitive decline after 12 months of treatment in patients with AD [14]. The largest study [1], involving millions of people under the care of the Veterans Administration, showed that simvastatin reduced levels of dementia and Parkinson’s disease by 50%.

An assay system has been developed to the study effects of various compounds on Aβ42 in the convenient model eukaryote, *Saccharomyces cerevisiae* [15]. This system offers a means of visualising Aβ42 in cells by fusing it to green fluorescent protein (GFP). The expression system utilises yeast plasmids that enable the constitutive production of GFP fused to Aβ42 in all cells. Yeast transformed with such plasmids are stably maintained in minimal selective media. 

In cells producing GFP fused to Aβ42, green fluorescence is observed in a small fraction of the cells (12–30%). In non-fluorescent cells the GFP fused to Aβ42 is absent, having been produced constitutively but then degraded [15]. The GFP-Aβ is deleterious to cells and causes a growth rate that is 95% of the normal rate [16]. This expression system can be used for assays to identify compounds that aid the clearance of GFP-Aβ and reduce green fluorescence. Assays are fairly straightforward and are usually performed with fixed amounts when compounds have no substantial effect on growth. However, the statins and azoles can substantially affect growth [17]. Therefore, the assay of these compounds in this system requires careful approaches and considerations. 

## 2. Results

### 2.1. Older Cells Do Not Degrade Aβ42 Fused to GFP Effectively

The expression systems to produce GFP fused to Aβ42 are constitutive, so all cells in the population containing the pAS1N.Aβ-GFP and p416GPD.GFP-Aβ plasmids are expected to produce Aβ-GFP or GFP-Aβ. However, as previously reported, only a small fraction of the population is fluorescent and contains GFP fused to Aβ42 [15,16]. Calcofluor staining and fluorescent microscopy previously showed that young cells degrade the GFP fused to Aβ42 and are not fluorescent while older cells retain the fusion protein and are fluorescent [15]. In this study, cells have been analyzed by Coulter flow cytometry which measured the diameter of cells as well as green fluorescence. As yeast cells age, they progressively get slightly larger.

**Figure 2:**
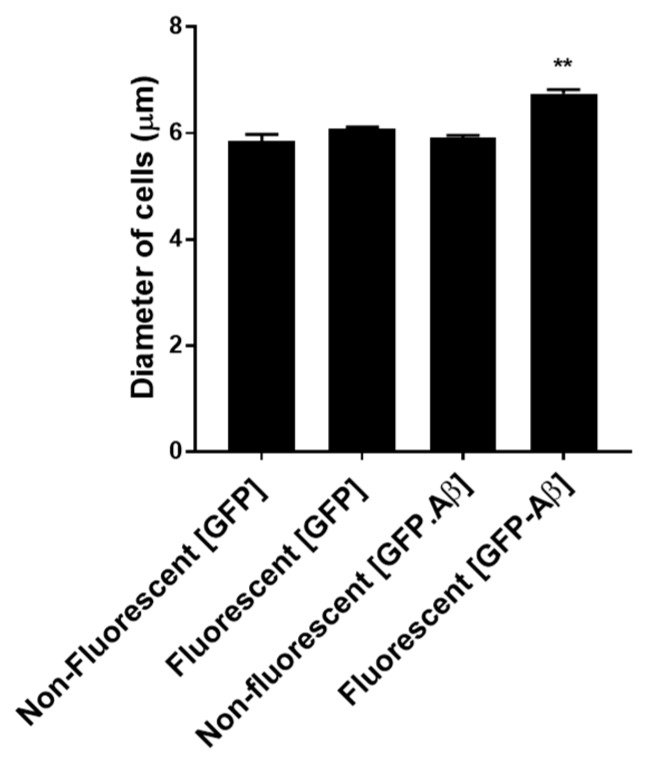
Analysis of cell diameters in cultures of BY4743 [p416GPD.GFP-Aβ] (indicated as [GFP-Aβ]) and BY4743 [p416GPD.GFP] (indicated as [GFP]) cells. Data were analyzed using Wilcoxon Rank Sum test and statistically significant values were indicated by asterisks above the bar (** p < 0.01).

A more quantitative demonstration that these older cells retain GFP fused to Aβ42 is shown in Figure 2 where the diameters of cells in different groups from cultures of BY4743 [p416GPD.GFP-Aβ] and BY4743 [p416GPD.GFP] have been compared. The average cell diameter is about 6.0 μm in the first three populations depicted: the minor population of non-fluorescent and the major population of fluorescent cells from BY4743 [p416GPD.GFP] (first two bars), and the non-fluorescent cells from BY4743 [p416GPD.GFP-Aβ] (third bar). There are no significant differences between these populations regarding cell size. However, green fluorescent cells of BY4743 [p416GPD.GFP-Aβ] (fourth bar), have a highly significant (*p* < 0.01) larger size of 6.7 µm. These are older cells that are a small proportion of the population and can also be distinguished by the numbers of bud scars (not shown). It is this older population that are the focus in this study. This population is a relatively small fraction (~12%). 

### 2.2. Statins Reduce Levels of Aβ42 Fused to GFP 

**Figure 3:**
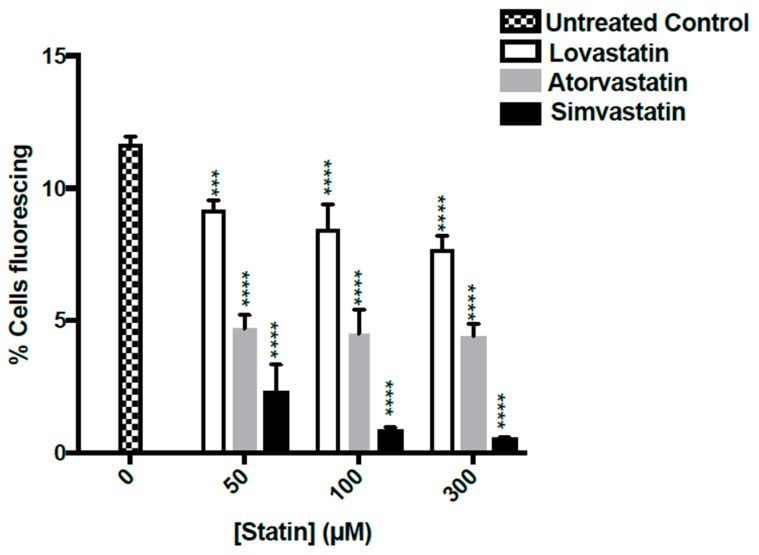
Effect of statins on proportion of green fluorescent cells. BY4743 [p416GPD.GFP-Aβ] cells were grown for 2 h with lovastatin (white bars), atorvastatin (grey bars) and simvastatin (black bars). After this treatment, the proportion of green fluorescent cells was determined by flow cytometric analysis. Values are from triplicates: values significantly different from the untreated controls in two-way ANOVA are indicated by asterisks above each bar (*** *p* < 0.001; **** *p* < 0.0001).

The present study was carried out to assess the effect of lovastatin, simvastatin and atorvastatin, on Aβ42 fused to green fluorescent protein expressed in the yeast. During two hours of statin treatments there was no significant toxicity since propidium iodide stained less than 1.5% of the population, regardless of statin treatment (see Figure S1). In this period there is no loss of mitochondrial DNA: mitochondrial DNA loss is only substantial when statin treatments continue for a day or more [17]. However, there were highly significant effects on the proportions of the green fluorescent cells (Figure 3).

In the untreated population, green fluorescent cells were ~12% of the population. Treatments with 50 μM simvastatin led to an ~70% reduction in green fluorescent cells. Treatments with 100 μM simvastatin led to a greater decrease in fluorescence and 300 μM simvastatin led to an even greater decrease: with 300 μM simvastatin only ~0.58% cells remained fluorescent.

Atorvastatin, at levels of 50, 100 and 300 μM, led to an ~60% decrease in green fluorescence, while the effects of lovastatin at these levels caused an ~20% reduction in green fluorescent cells. 

These studies demonstrate that in a population of exponentially-growing yeast cells statins can aid the clearance of GFP-Aβ from the major proportion of older cells. Simvastatin caused the greatest clearance, followed by atorvastatin and then lovastatin.

### 2.3. Is Reduction of Aβ Fused to GFP Related to Inhibition of Ergosterol Synthesis?

Statin-induced reduction of GFP-Aβ could be a consequence of lowering ergosterol (the yeast cholesterol equivalent) where there are reduced opportunities for membrane localisation of the hydrophobic GFP-Aβ. This could make GFP-Aβ more available for proteolytic clearance.

**Figure 4:**
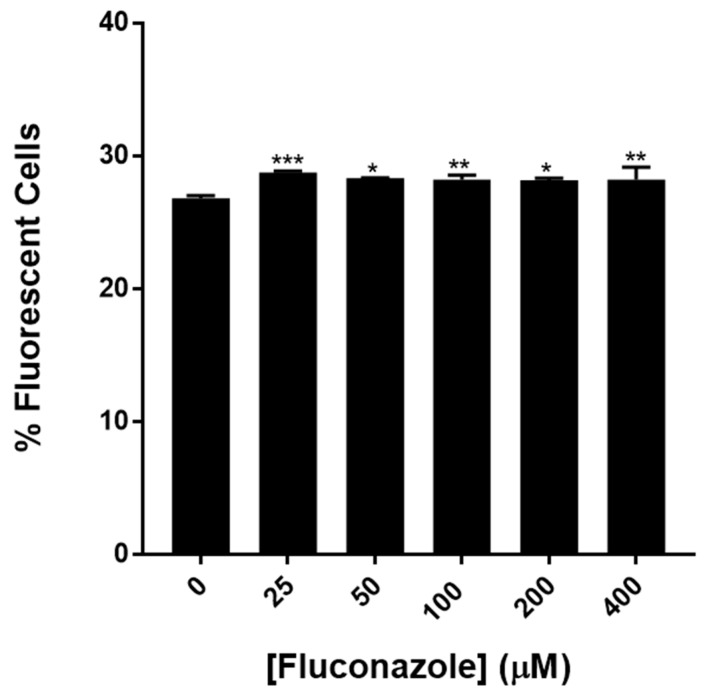
The effect of fluconazole treatment on the proportion of green fluorescent cells in a population of BY4743 [pAS1N.Aβ-GFP] cells cultured for 2 h with fluconazole at the levels indicated. Values are from triplicates and values significantly different from 0 fluconazole in one-way ANOVA are indicated by asterisks (* *p* < 0.05; ** *p* < 0.01; *** *p* < 0.001).

In order to examine effects related to ergosterol biosynthesis experiments were performed using fluconazole, an antifungal drug that reduces ergosterol biosynthesis. The IC50 (concentration at which fluconazole caused 50% growth inhibition) was 93 μM: Concentrations above and below this level were tested for their effects on the population (see Figure 4). 

Fluconazole treatment of BY4743 [pAS1N.Aβ-GFP] resulted in small increases in the population of fluorescent cells. The increases with 25–400 μM fluconazole were statistically significant. Increases were between 5.09% and 7.26% compared to the untreated control. These increases are not due to fluconazole causing cells to fluoresce independent of GFP content, as fluconazole treatment on non-transformed cells did not result in increased fluorescence (see Figure S2). A possible mechanism explaining increased fluorescence under fluconazole treatment is for the GFP fused to Aβ42 being unable to associate with the plasma membrane due to reductions in membrane ergosterol levels. The resulting accumulation of the GFP fused to Aβ42 in the cytosol may result in increased cell fluorescence.

### 2.4. Expression of Native Aβ42 Using pYEX-BX 

**Figure 5:**
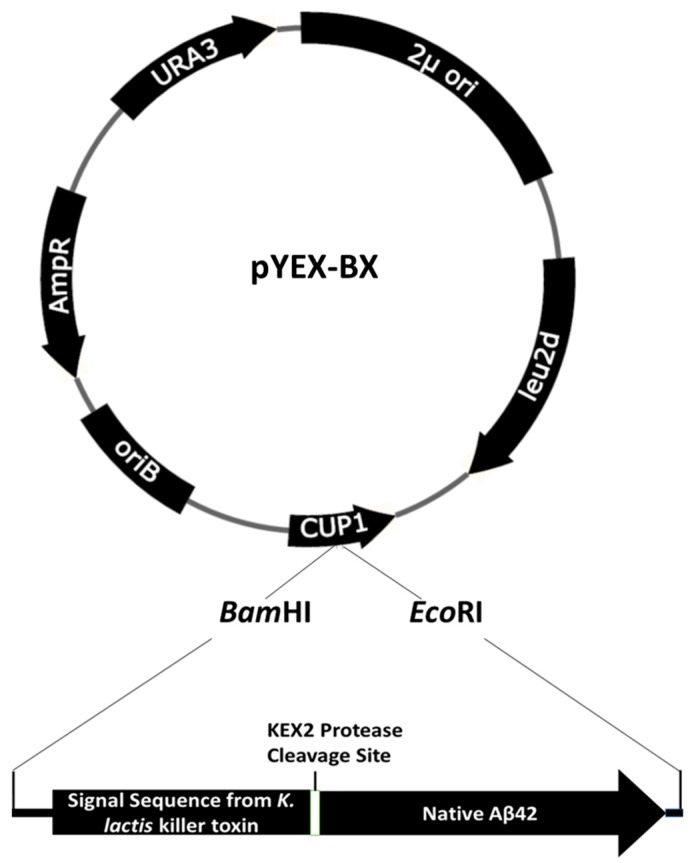
Schematic diagram of the pYEX-BX plasmid along with the insert enabling production of native Aβ42.

In order to examine how simvastatin affects turnover of native Aβ42, sequences encoding the native Aβ42 fused to a secretion signal peptide were introduced into the yeast expression vector, pYEX-BX (Figure 5, previously named pYEULCBX [18]). The secretion signal peptide is designed to be cleaved by the yeast KEX2 protease, so after cleavage native Aβ42 is produced. The insertion was made at the *CUP1* locus, which enables copper-inducible expression of foreign genes. Possible complications of copper induction and toxicity were avoided by not using copper as an inducer. Instead our work utilised expression of *CUP1* promoter at its basal level (which is around 4% of the copper induced levels). In the absence of leucine there is selection for increased copies of the *leu2d* gene, which enables a higher number of plasmid copies and higher expression levels of foreign protein (discussed in [19]). MALDI-TOF mass spectrometry confirmed the production of native Aβ42 of 4515.751 daltons.

### 2.5. Simvastatin Reduces Levels of Native Aβ42 Expressed in Saccharomyces cerevisiae

**Figure 6:**
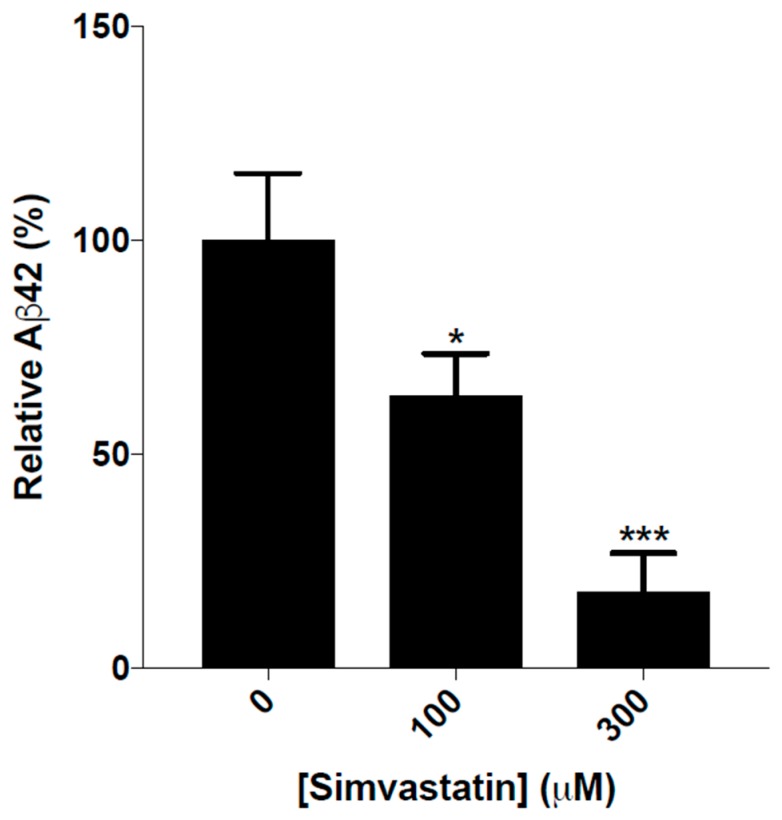
Relative amounts of Aβ42 in BY4743 [pYEX-Aβ] cells treated with 0, 100 and 300 µM simvastatin. Experiments were performed in triplicate. Statistical analyzes were performed using one-way ANOVA tests and significant values in comparison with untreated controls are indicated by the asterisks above the bar (* *p* < 0.05; *** *p* < 0.001).

BY4743 [pYEX.Aβ] transformants were used to quantitate the levels of native Aβ42 in response to simvastatin treatment. Cellular fractionation showed that the Aβ42 was associated with the membrane fraction, so this was the portion that was analyzed by quantitative MALDI-TOF protein mass spectrometry. The levels of Aβ42 were normalized relative to the amount of cells. The reduction in levels of Aβ42 was found to be statistically significant for the cells treated with varying concentrations of simvastatin. Figure 6 depicts the differences in the relative levels of Aβ42 detected in different cell samples. The data used to produce Figure 6 can be found in Figure S3, Table S1 and S2. The 300 µM simvastatin concentration was found to be highly effective in reducing the levels of Aβ42.

## 3. Discussion

This study shows the utility of examining the levels of native Aβ42 and Aβ42 fused to GFP where it can be observed in living cells. The study showed that young cells of yeast have efficient clearance of the deleterious Aβ42 fused to GFP, but that older yeast cells exhibit low or no turnover of this protein. Furthermore, this is the first report to show that statins aid in reducing levels of Aβ42 fused to GFP and native Aβ42 expressed in yeast, and we favour the idea that statins can affect clearance of Aβ42. 

Lower levels of Aβ42 fused to GFP, after 2 hours of treatment with statins, could be observed in a portion of the older cells. The effect was greatest with simvastatin, followed by atorvastatin and then lovastatin. The reductions were highly statistically significant and dose-dependent at treatments ranging from 50 to 300 μM. Statins can cause some major effects on yeast. By decreasing ergosterol levels in prolonged cultures statins cause growth inhibition and this also can cause total loss of the mitochondrial genome [17,20]. For this reason, our statin treatment times were for 2 hours. During this time cells remained healthy, exhibiting no killing and there was no loss of mtDNA [17]. 

As a comparison, the effects of fluconazole, which also affects the ergosterol biosynthesis pathway, were also investigated. Fluconazole did not reduce levels of Aβ42 fused to GFP: In fact, there was a slight increase. This suggests that the statins are exerting most of their effects by targeting a protein clearance pathway. There are several pathways involved in protein clearance and some of them have previously been shown to be affected by statins. Protein clearance mechanisms affected by simvastatin, lovastatin and atorvastatin include autophagy and the unfolded protein response [21]. The drug latrepirdine has some support for treatment of AD and also been found to reduce levels of Aβ42 fused to GFP in yeast by the autophagy pathway [22]. 

Our studies show that ergosterol levels on their own are not a major factor in clearance of Aβ fused to GFP. Indeed, Aβ-GFP expressed in a library of ~4600 *S. cerevisiae* mutants, identified 110 gene deletant mutants with increased green fluorescence [23]. Approximately 8% of those mutants showed defects in phospholipid homeostasis. A previous study [24] has shown that phytosterols can modify levels of Aβ42, however, it is most likely that they do this through modification of the membranes where processing of Aβ42 from the Amyloid Precursor Protein (APP) takes place.

Despite the convenience of studying GFP fused to Aβ42, there are expected to be differences to native Aβ42. Therefore, this study also expressed native Aβ42 in yeast using a new construct, pYEX.Aβ. The expression construct contains the signal sequence of *K. lactis* killer toxin with the KEX2 cleavage site (KR residues) at the C-terminus followed by the Aβ42 sequences, which results in the production of Aβ42 within the cells. The production of native Aβ42 was confirmed by MALDI-TOF mass spectrometry. Importantly, the use of yeast systems to produce native Aβ42 and Aβ42 fused to GFP provides platforms for assays to identify drugs and chemicals that may have anti-amyloidogenic properties.

In this study, yeast producing native Aβ42 has been used to examine the effect of simvastatin in the clearance the 42 amino acid protein. The reduction in the levels of native Aβ42 after simvastatin treatment show that the simvastatin aids the clearance native Aβ42 inside yeast.

Statins have been associated with lower levels of Aβ42 in mammalian cell culture [25,26] and this has been attributed to effects on the prenylation of BACE, one of the enzymes involved in the cleavage of Aβ from the APP [15,27]. The current study is unique in that Aβ42 is produced independently of protein prenylation and protein clearance is proposed to be a major factor. Clearance of Aβ42 by statins is expected to reduce the risk of AD, by inducing a protein clearance response in the cells with the accumulated Aβ toxic proteins.

It is worth noting that in the epidemiology study of Wolozin et al. simvastatin stands out above other statins, including atorvastatin and lovastatin as preventing dementia [1]. In our study also, simvastatin was more effective than atorvastatin and lovastatin in clearing Aβ42 fused to GFP. Another reason why simvastatin is more protective in people may be due to it being the most lipophilic statin, possibly aiding greater entry to the brain. We have not found information pertaining to statin levels in brain. In contrast, factors involving bioavailability in humans are less relevant to yeast where all cells are exposed.

## 4. Materials and Methods

### 4.1. Yeast Strains and Growth

The *Saccharomyces cerevisiae* yeast strains BY4743 (*MATa*/α *his3*Δ1/*his*3Δ1, *LYS2*/*lys2*Δ0 *met15*Δ0/*MET15 ura3*Δ0/*ura3*Δ0 *leu2*Δ0/*leu2*Δ0) was the host strain used in this study. The plasmids pAS1N.Aβ-GFP, pAS1N.GFP, p416GPD.GFP-Aβ, p416GPD.GFP and pYEX-Aβ were transformed into the host strain as described by Porzoor and Macreadie [15]. The Aβ42 used in this study is the full-length peptide, composed of 42 amino acids [15]. BY4743 [pAS1N.Aβ-GFP] and BY4743 [p416GPD.GFP-Aβ] were used interchangeably in this study and give similar trends in results. Minimal selective media was used for growth of the BY4743 transformants. The media composition was as follows: Yeast nitrogen base without amino acids (0.67%), dextrose (2%) and agar (1.5%) (if solid media was required). Supplementation of auxotrophic requirements was performed by adding leucine 20 mg/L, histidine 20 mg/L and uracil 20 mg/L, where required.

### 4.2. Hydrolysis of Statins

Two statins, lovastatin and simvastatin were hydrolyzed to activate them from inactive lactone form to active hydroxyl acid form [28,29]. Atorvastatin does not require pre-activation by hydrolysis. The prodrugs lovastatin (10 mg) and simvastatin (10 mg) were hydrolyzed in 1 mL of ethanolic NaOH [15% (*v/v*) ethanol and 0.25% (*w/v*) NaOH] at 60 °C for 60 min. The stock solutions of hydrolyzed statins were stored at −20 °C. 

### 4.3. Effect of Statins and Fluconazole on Yeast Cells

Yeast cells were analyzed for effect of all three statins and fluconazole on toxic Aβ42 protein clearance. Overnight fresh cultures of yeast cells were obtained by inoculating 3–4 colonies on fresh selective minimal media in a 15 mL centrifuge tube. The tubes were incubated at 30 °C at 200 rpm. Overnight cultures were further grown to exponential phase in order to achieve high levels of fluorescence. The cell culture was obtained after inoculating 1 mL of overnight culture into 4 mL of fresh minimal media in centrifuge tubes. The tubes were incubated at 30 °C and 200 rpm for 2 h. These cells were centrifuged for 2 min at 252× *g* after 2 h. Centrifuged cell pellets were resuspended into 1 mL selective minimal media broth. Statins were added at a final concentration 50–300 μM while fluconazole was added at concentrations up to 400 µM. Incubations were performed at 30 °C and 200 rpm for 2 h. Flow cytometry was performed on strains treated with statins and fluconazole.

### 4.4. Flow Cytometry

Flow cytometry was done to determine the number of cells with green fluorescence. Flow cytometry was performed using FACScan flow cytometer (BD FACS CantoTM II, Becton-Dickinson, Franklin Lakes, NJ, USA). The data acquisition was done using BD FACSDiva Software. For each sample, 10,000 cells per sample were analyzed (*n* = 3). Flow cytometry utilised excitation filters with 488 nm (blue laser) as the GFP protein excites in the wavelength ranging from 400–530 nm. Fluorescence emission was measured at 530/30 emission filters. 

### 4.5. Native Aβ42 Vector Construction

The native Aβ42 expression vector was constructed using the yeast optimized gene construct encoding a secretion signal (*Kluyveromyces lactis* killer toxin) preceding Aβ42 in the construct. The synthetic construct was made by GeneArt and cloned into their vector. A *Bam*HI-*Eco*RI double digest liberated the desired fragment which was cloned in the *Bam*HI-*Eco*RI digested target vector, pYEX-BX. The resulting construct, shown in Figure 5 is pYEX-Aβ. The pYEX-Aβ plasmid was verified by DNA sequencing.

### 4.6. Transformation of Plasmids into S. cerevisiae

*S. cerevisiae* BY4743 cells were transformed using the EZ transformation kit. Isolated colonies of 3–4 mm diameter from freshly grown YEPD plates were dissolved in 125 µL EZ transformation solution. Cells were re-suspended, and to the suspension was added with 5 µL of carrier DNA (Salmon Sperm) and 2 µg plasmids. The solution was vortexed and incubated at 42 °C for 30 min. Following the incubation, the mixture was then transferred into solidified minimal media lacking uracil or leucine for selection of the transformants. The plates were incubated at 30 °C for 2–10 days. A negative control and empty vector transformations were also performed alongside to validate the transformation. Transformed yeast were checked for the correct phenotype.

### 4.7. Quantitative MALDI-TOF Mass Spectrometry to Detect the Levels of Aβ42

#### 4.7.1. Growth of Transformants Expressing Native Aβ42

The BY4743 [pYEX-Aβ] transformants expressing native Aβ42 were grown overnight in minimal media containing 20 mg/L histidine at 30 °C. Next day the yeast cells were supplied with fresh media and re-grown for 2 h. The cells were then grown for a further 2 h with simvastatin concentrations of 0, 100 and 300 µM. Cultures were performed in triplicate.

#### 4.7.2. Sample Preparation for MALDI-TOF

Simvastatin-treated cells were harvested from liquid culture by centrifugation at 3000 rpm for 3 min. Cells were washed two times in TBS and resuspended in 500 µL TBS, followed by cell disruption in an MP Fast tissue homogenizer using glass beads (0.5 mm). Six cycles (1 minute each cycle) of homogenization were performed with a resting period of 1 minute in cold ice between each consecutive cycle of disruption. The lysate was transferred into sterile clean Eppendorf tubes and were fractionated using differential centrifugation. Large cell debris were separated by centrifuging the cells at 300 rpm for 3 min and the fraction of insoluble proteins from the supernatant was collected by centrifuging the supernatant at 15,000 rpm for 10 min. The insoluble pellet fraction was then added with 25 µL of TBS buffer and kept on ice until further processing. 

#### 4.7.3. Measurement of Relative Quantity of Aβ42 Using MALDI-TOF

The measurement of Aβ42 using MALDI-TOF mass spectrometry in this study was similar to the one described previously [30] with a few changes to suit the demands of the study. Bruker MALDI TOF has been used to measure the amount of protein in the mixture of proteins. The insoluble fraction of the cells treated with different concentration of simvastatin were spotted onto the MALDI plate (anchorchip). To every sample spot was added an equal amount of BTS internal calibration standard of known molecular weight. To overcome suppression of less abundant Aβ42 ions, 1 µL of 1 µM synthetic Aβ42 (Keck laboratories, Yale University, New Haven, CT, USA) was added to increase the signal. The synthetic Aβ42 is identical to native Aβ42 and has the same m/z values. After drying the plates, each sample spot was flooded with 2 µL of MALDI matrix HCCA. The spots were air dried so that the samples could co-crystallize with the matrix. A standard curve was generated, in the same way, using the non-transformant *S. cerevisiae* insoluble protein extract as a background. Known amounts of the synthetic Aβ42 were added into the spots to generate a standard curve. For calibration, each of these spots were also added with the same amount of internal calibrant used for the samples. Data from the plate was collected manually using the Flex Control software, which controls the MALDI-TOF mass spectrometer. The data obtained were normalized according to the peak height of the internal calibration standards. Using the standard curve, the relative amount of the Aβ42 in three different samples were calculated and analyzed. The statistical software GraphPad Prism Version 7 has been used to analyze the data collected. 

## 5. Conclusions

Statins have the potential to reduce the risk of AD, by inducing a protein clearance response in the cells. More extensive research in yeast can be done to decipher the mechanism of actions of statins within the eukaryote and to screen for compounds that might have more significance than simvastatin in clearing Aβ42 fused to GFP or native Aβ42 from the cells. The use of statins for the treatment of other neurodegenerative diseases such as Parkinson’s Disease, Huntington’s Disease and prion diseases should be investigated in the future for their application as therapeutic agents.

It is also important to confirm this statin-induced clearance of Aβ42 in studies involving human cells, however, this may require producing Aβ42 independently of APP, to avoid the effects of statins on BACE prenylation. 

## Supplementary Materials

The supplementary materials are available online at https://www.mdpi.com/1422-0067/20/14/3531/s1.

## Abbreviations

AD	Alzheimer’s disease
Aβ42	β-amyloid of 42 amino acids
BACE	Beta-secretase enzymes
DMSO	Dimethyl sulphoxide
GFP	Green fluorescent protein
HCCA	2-Cyano-3-(4-hydroxyphenyl) acrylic acid
IPOD	Insoluble protein deposit
MALDI	Matrix Assisted Laser Desorption Ionization
PBS	Phosphate buffered saline
TBS	Tris buffered saline
TBS-T	Tris buffered saline with 0.05% Tween 20
TOF	Time of flight
μM	Micromolar
