# Supplementary material for: Simvastatin Efficiently Reduces Levels of Alzheimer’s Amyloid Beta in Yeast
Source: Int J Mol Sci. 2019 Jul 19;20(14):3531. doi: 10.3390/ijms20143531 (PMC6678968; doi:10.3390/ijms20143531)
Supplement: Supplementary file 1 [file ijms-20-03531-s001.pdf]

Supplementary files

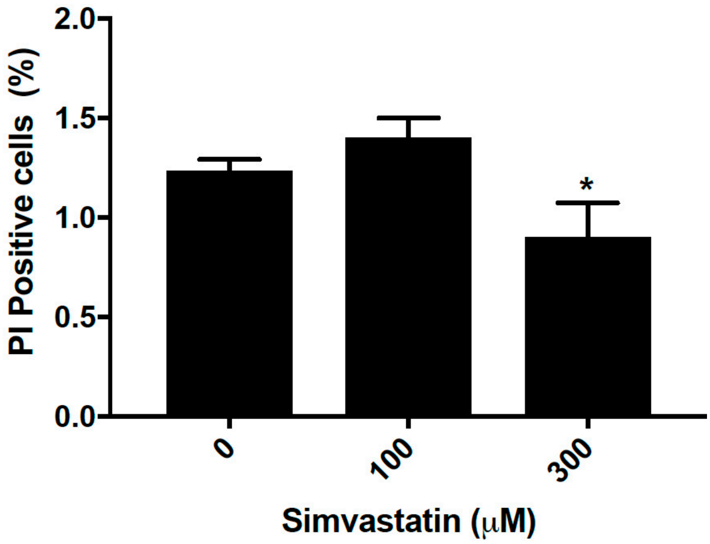

Figure S1. There is no significant toxicity induced by simvastatin in 2 hours.

BY4743 cells were grown overnight and then re-grown for 2 hours with 0, 100 and 300 μM simvastatin before being stained with 0.5 μg/ml propidium iodide. A population of 10,000 cells were analysed in triplicate by flow cytometry to measure red fluorescent cells. Statin treatment caused no increased cell death.

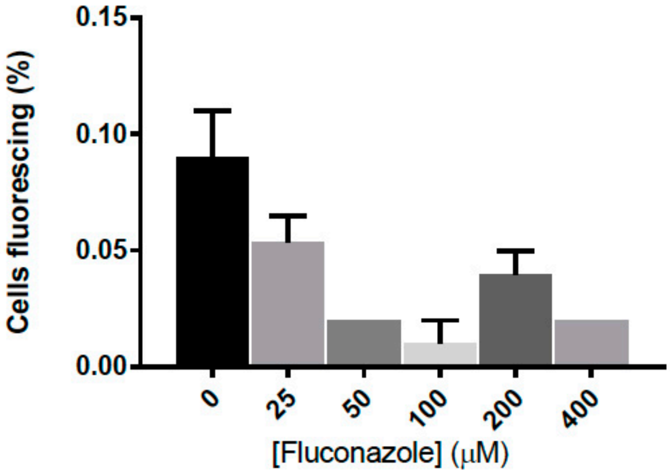

Figure S2. Fluconazole does not cause increased green fluorescence.

BY4743 cells were grown overnight and then re-grown for 2 hours up to 400 μM fluconazole. A population of 10,000 cells were analysed in triplicate by flow cytometry to measure green fluorescent cells. Statin treatment caused no increased green fluorescence.

Information relating to Figure 6

Determination of Standard Curve for estimation of Aβ42 in unknown samples

Saccharomyces cerevisiae BY4743 [pYEX-BX] transformants were grown and harvested as described in the methods 4.9. Samples of the insoluble portion of the cell lysate were collected and spotted onto four spots in the anchorchip plate used for MALDI TOF measurement to normalize the background noise comparable to the samples in the study. To each spot was added 4.5 to 11.2 ng amounts of synthetic Aβ42 (serially diluted from a 5 µM stock), internal calibrant and 2 µL of matrix HCCA. The sample spots were then analysed using a Bruker MALDI TOF mass spectrometer. The corresponding mass to charge (m/z) peak height for each concentration of Aβ42 were measured and used to determine a standard curve (peak height vs amount of Aβ42) for calculation of Aβ42 from the samples with unknown quantities of Aβ42.

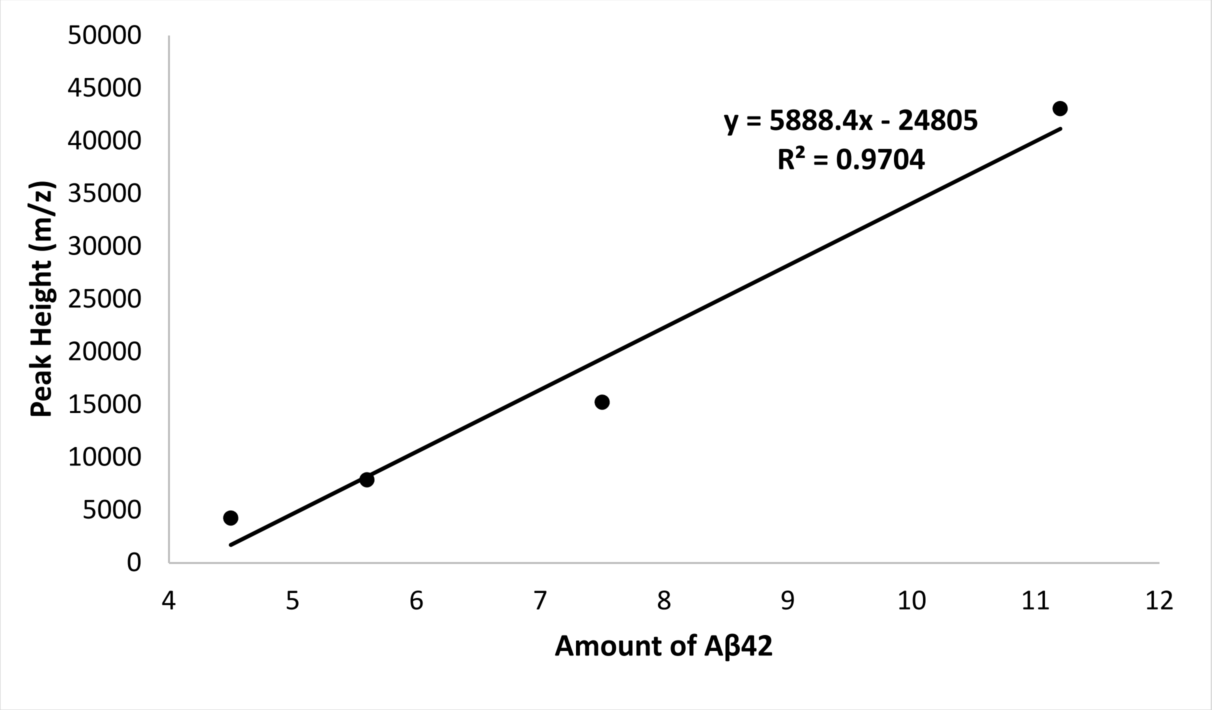

Figure S3. Standard Curve obtained after plotting peak height of mass to charge ratio versus amount of Aβ42 in a scatter plot for determination of amount of Aβ42 in different samples.

Calculation of Aβ42 from the peak heights of the samples with unknown amount

For preparation of yeast fractions for mass spectrometry quantitation refer to 4.8 in the main manuscript. The relative amounts of Aβ42 in each sample was estimated using the standard curve obtained above. The amounts of Aβ42 are shown in the last column of Table S1, and these amounts are normalised (Table S2) and that information is depicted in Figure 6 of the manuscript.

Table S1. Calculation of Aβ42 amounts in the samples treated with different concentrations of simvastatin

Sample	Simvastatin (µM)	Replicate	Volume of 1µM Aβ42 added in each spot (µL)	Peak Height of mass to charge ratio	Amount of Aβ42 (ng)	Net amount of Aβ in each sample (ng)
**A**	0	1	1	17801	8.45	2.72
		2		13456	7.15	1.98
		3		15642	7.80	2.35
**B**	100	1	1	9109	5.84	1.24
		2		10949	6.39	1.55
		3		11752	6.63	1.69
**C**	300	1	1	5362	4.72	0.60
		2		4463	4.45	0.45
		3		2887	3.97	0.18

Relative changes of the Aβ42 in different samples are represented in the following Table S2.

Table S2. Relative change in Aβ42 in different samples

Sample	Replicate	Net amount of Aβ42 in different samples	Average	Percentage turnover of Aβ42 in each sample
**A**	1	2.72	2.35	115.6
	2	1.98		84.2
	3	2.35		100.0
**B**	1	1.24	1.49	52.9
	2	1.55		66.1
	3	1.69		71.9
**C**	1	0.60	0.41	25.8
	2	0.45		19.3
	3	0.18		8.0
